# *Curtobacterium uvaldense* sp. nov., *Falsirhodobacter allii* sp. nov., *Pseudomonas texasensis* sp. nov., Three Novel Species Isolated from Onions in Texas, USA

**DOI:** 10.1007/s00284-026-05144-1

**Published:** 2026-09-09

**Authors:** Manzeal Khanal, Bed Prakash Bhatta, Lakhvir Kaur, Subas Malla

**Affiliations:** 1 Department of Horticultural Sciences, Texas A&M University, College Station, TX 77843 USA; 2 Texas A&M AgriLife Research and Extension Center, Uvalde, TX 78801 USA; 3 Present Address: Department of Environmental Science, University of Arizona, Tucson, AZ 85719 USA; 4 Present Address: Department of Agriculture, Alcorn State University, Lorman, MS 39096 USA

## Abstract

Bacterial strains 20TX0008^T^ and 20TX0035^T^ were isolated from the symptomatic onion foliage in 2020, and a bacterial strain 21TX0197^T^ was isolated from the symptomatic onion bulb in 2021. Pathogenicity assays confirmed the two strains isolated from foliage to be non-pathogenic, while the strain 21TX0197^T^ was found to be pathogenic. Phylogenetic analysis using the 16 S rRNA gene revealed the closest resemblance of 20TX0008^T^, 20TX0035^T^, and 21TX0197^T^ to the genera *Curtobacterium*, *Falsirhodobacter*, and *Pseudomonas*, respectively. For the respective genus, strains 20TX0008^T^ showed the greatest 16 S rRNA sequence similarity with *Curtobacterium caesinilyticum* GDMCC 1.2667^T^ (99.7%), 20TX0035^T^ with *Falsirhodobacter deserti* W402^T^ (99.1%), and 21TX0197^T^ with *Pseudomonas uvaldensis* 20TX0172^T^ (99.4%). In the phylogenetic trees based on whole genome sequences, all three strains formed a distinct, well-supported branch that separated them from their closely related species within the same genus. The genomic similarity, based on average nucleotide identity (ANI) and digital DNA-DNA hybridization (dDDH), revealed that the novel strains had values below the species delineation thresholds (ANI < 95% and dDDH < 70%), confirming the novelty of all these strains. Based on genomic and phenotypic characteristics, we propose three novel species, *Curtobacterium uvaldense* sp. nov. (type strain 20TX0008^T^ =LMG 33207^T^ =NCIMB 15480^T^), *Falsirhodobacter allii* sp. nov. (type strain 20TX0035^T^ =LMG 33208^T^ =NCIMB 15477^T^), and *Pseudomonas texasensis* sp. nov. (type strain 21TX0197^T^ =LMG 33210^T^ =NCIMB 15479^T^).

**Supplementary Information:**

The online version contains supplementary material available at https://doi.org/10.1007/s00284-026-05144-1.

## Introduction

Bacteria are among the most diverse and rapidly evolving organisms, constantly adapting to changing environments and developing new traits that allow them to survive and thrive [1]. This adaptability drives the emergence of new species that have a substantial impact on agriculture, human health, and various ecosystems. The identification and characterization of these rapidly evolving species are essential for environmental sustainability and the development of effective disease management practices. Breakthrough advancements in nucleic acid sequencing technologies have made the identification and characterization of novel species easier, faster, and cost-effective [2]. Genomic analyses such as average nucleotide identity (ANI) and digital DNA-DNA hybridization (dDDH) consider the entire genome and are highly effective in differentiating closely related species [3, 4].

*Curtobacterium* is a genus of bacteria belonging to the phylum *Actinomycetota* (class–*Actinomycetia*, order–*Micrococcales*), and bacteria in this genus are Gram-positive, obligately aerobic, non-spore-forming, and catalase-positive [5]. As of December 2025, it comprises a total of 14 species (LPSN, https://lpsn.dsmz.de/genus/curtobacterium) with nomenclature recognized as validly published and correct under the International Code of Nomenclature of Prokaryotes (ICNP) [6]. Of these, only two species, *Curtobacterium flaccumfaciens* and *C. allii* are known to be plant pathogenic [7, 8]. While some species such as *C. albidum*, *C. citreum*, and *C. luteum* were isolated from rice plants, *C. allii* was isolated from an onion bulb [9].

*Falsirhodobacter* is a genus of bacteria belonging to the phylum *Pseudomonadota* (class–*Alphaproteobacteria*, order–*Rhodobacterales*), and bacteria in this genus are Gram-negative, obligately aerobic, non-spore-forming, and catalase-positive. As of December 2025, it comprises a total of 4 species (LPSN) with nomenclature recognized as validly published and correct under the ICNP [6]. Of these, one was isolated from a dry soil sample [10], one from a rhizosphere soil sample [11], one from a seawater sample [12, 13], and one from red algae [14]. Until now, no species of this genus had been isolated from plant tissue.

*Pseudomonas* is a genus of bacteria belonging to the phylum *Pseudomonadota* (class–*Gammaproteobacteria*, order–*Pseudomonadales*), and bacteria in this genus are Gram-negative, obligately aerobic, non-spore-forming, and catalase-positive. As of December 2025, it comprises a total of 362 species (LPSN) with nomenclature recognized as validly published and correct under the ICNP [6]. Until now, there are at least 8 species of this genus that are pathogenic to onion, including *Pseudomonas uvaldensis*, which was isolated recently from Texas, USA [15].

The current study aimed to isolate, identify, and characterize novel bacterial species associated with onions, focusing on their physical and physiological properties to expand the growing database of bacterial diversity as well as to support the development of effective disease management practices in onions. A polyphasic approach combining phenotypic and genotypic analyses of bacterial strains collected during the onion disease survey led to the identification of three novel species [16], as described below.

## Materials and Methods

### Isolation and Ecology

Two strains, 20TX0008^T^ and 20TX0035^T^, were isolated from symptomatic onion foliage tissue in 2020, and strain 21TX0197^T^ was isolated from the symptomatic onion bulb tissue in 2021, from onion cultivated in Texas, USA. Bacterial isolation along the margin of the symptomatic tissue of foliage or bulb was done on nutrient agar (NA) medium as previously described [16]. Briefly, a 5 mm piece of symptomatic tissue was removed, washed, and macerated in 100 µl of sterile water. Following the streaking of the resulting suspension on nutrient agar (NA) medium, a single colony was purified on NA and incubated at 25 °C. The isolated single colonies were further sub-cultured at the same temperature until a pure culture was obtained and then stored in sterile 15% aqueous glycerol at − 80 °C. Pathogenicity of the purified bacterial isolates was confirmed via a red scale necrosis assay, a bulb assay, and a foliar assay with the positive (*Pantoea ananatis* strain PNA 97-1R) and negative (phosphate-buffered saline) controls, as previously described [16]. The scale assay used wounded scales (~ 3 × 4 cm) of fleshy red onion bulbs for the inoculation of 10 µl bacterium suspension of the isolated species, which was then incubated at 25 °C for 4 days. For the bulb assay, a bacterial suspension of the isolated species (0.5 ml) was injected into the upper shoulder of the yellow onion bulb, followed by incubation at 25 °C for 12 days. For the foliar assay, leaves of 5-week-old plants were cut 7 cm from the tip and inoculated at the top using a 20 µl bacterium suspension of the isolated species, followed by incubation at 25 °C for 5 days. Each assay was conducted three times, and the whole experiment was repeated twice.

### 16 S rRNA Gene Phylogeny

Genomic DNA of strains 20TX0008^T^, 20TX0035^T^, and 21TX0197^T^ was extracted using DNeasy PowerSoil Kit (Qiagen) and Sanger sequenced using universal primers 27F and 1492R to determine the sequence of the 16S rRNA gene [16, 17]. Sequences derived from two primers were trimmed and assembled to make a consensus sequence using Geneious Prime 2020 (https://www.geneious.com/). The partial 16S rRNA sequences of 20TX0008^T^, 20TX0035^T^, and 21TX0197^T^ were deposited in GenBank with accession numbers ON255719, ON255745, and OP325568, respectively. These sequences were compared with the closely related species (Reference Sequence) using the NCBI Basic Local Alignment Search Tool (BLAST) [18]. Maximum-likelihood phylogenetic trees based on the closely matching type strains were constructed using MEGA 12 [19], with 1000 bootstrap replicates and the Tamura-Nei model [20].

### Genomic Analysis and Functional Annotation

The genomic DNA of the isolated strains was sent for whole-genome sequencing and assembly to SeqCenter (SeqCenter LLC, PA, USA). The sample libraries were prepared and sequenced on the Illumina NextSeq 2000 platform with an average sequencing depth of 100X, resulting in 2 × 151 bp paired-end reads. The raw fastq files were demultiplexed, quality controlled, and adapter trimmed using bcl-convert v.3.9.3 [21]. The trimmed reads were then assembled using SPAdes v.3.13.0 [22]. Finally, the genome quality of assembled reads was estimated by checkM2 (Galaxy Version 1.1.0 + galaxy0), and they were deposited in GenBank with genome accession numbers: JAQMHU000000000 (20TX0008^T^), JAQMHV000000000, (20TX0035^T^), and JAQMHS000000000 (21TX0197^T^).

Genome relatedness with the closely related species was calculated using average nucleotide identity (ANI) and digital DNA-DNA hybridization (dDDH) values (Table 1). The orthologous ANI (orthoANIu) algorithm (https://www.ezbiocloud.net/tools/ani) and ANIblast (ANIb) were used to calculate ANI values [23, 24], and dDDH values [25] were computed using Type Strain Genome Server (TYGS, https://tygs.dsmz.de/). Strains were designated as a novel species if ANI and dDDH values were < 95% and < 70%, respectively [26].

**Table 1:** Genomic similarities between each strain (*Curtobacterium uvaldense* 20TX0008ᵀ, *Falsirhodobacter allii* 20TX0035ᵀ, and *Pseudomonas texasensis* 21TX0197ᵀ) and their closely related species

Novel strain and closely related species	dDDH (d4, %)	OrthoANIu (%)	ANIb (%)	16 S rRNA (%)
***Curtobacterium uvaldense 20TX0008*** ^T^				
*Curtobacterium citri* GDMCC 1.2668^T^	40	86.9	86.8	99.4
*Curtobacterium caseinilyticum* GDMCC 1.2667^T^	34	87.6	87.7	99.7
*Curtobacterium subtropicum* GDMCC 1.2669^T^	32.1	90.1	90.2	99.4
***Falsirhodobacter allii*** **20TX0035**^**T**^				
*Falsirhodobacter deserti* W402^T^	23.3	80.9	79.8	99.1
*Falsirhodobacter algicola* PG104^T^	22.7	80.4	79.2	98.1
*Falsirhodobacter halotolerans* KCTC32158^T^	21.4	79.3	77.5	98.4
***Pseudomonas texasensis*** **21TX0197**^**T**^				
*Pseudomonas uvaldensis* 20TX0172^T^	35.7	88.4	87.9	99.4
*Pseudomonas mediterranea* CFBP 5447^T^	32.6	86.8	86.2	98.6

Phylogenetic trees were generated using a manually curated pipeline incorporating Prodigal, HMMER, and IQ‑TREE to identify single‑copy core genes, align and concatenate marker sequences, and infer the final tree. Protein coding sequences were predicted from the genomes of closely related strains using Prodigal v2.6.3 with default parameters. Predicted proteomes were searched against the Pfam-A profile hidden Markov model (HMM) database using HMMER v3.3.2 (hmmsearch) to identify conserved protein domains across genomes. For each genome, HMMER tabular output was parsed to quantify the presence and copy number of each Pfam domain. Pfam entries that were (i) detected in all genomes and (ii) present as a single copy per genome were retained as putative single copy core markers. For each such Pfam domain, the best scoring hit per genome was extracted from the corresponding proteome FASTA file and used to construct per marker multiple sequence alignments with MAFFT v7. Individual marker alignments were then concatenated into a supermatrix, which was used to infer a concatenated protein phylogeny with IQ-TREE v3.0.1 under a model selected by ModelFinder, and node support was assessed with ultrafast bootstrap resampling (1000 replicates). Additionally, phylogenetic trees were constructed based on the whole-genome sequences through TYGS web server, where intergenomic distances were calculated based on Genome Blast Distance Phylogeny method (GBDP). For each calculation, d5 formula and 100 bootstrap replications were conducted [27].

Genome annotation of the strains was done using the Rapid Annotation using Subsystem Technology (RAST) server (https://rast.nmpdr.org/) [28] and SEED Viewer (https://www.theseed.org/wiki/Home_of_the_SEED) [29]. RAST annotations provided functional roles for predicted genes, grouped into subsystems. For simplicity, we refer to these functional roles as ‘genes’ throughout the manuscript, unless otherwise specified.

### Physiological Features

Physiological and chemotaxonomic studies of the bacterial strains were carried out following the established laboratory protocols [30, 31]. The 24-h-old cultures of 20TX0008^T^, 20TX0035^T^, and 21TX0197^T^ were used to test for the Gram reaction and KOH test [32]. The endospore-forming ability, oxidase activity, catalase activity, and the KB fluorescence tests were performed using malachite green, 3% hydrogen peroxide, Oxistrips (Hardy Diagnostics, CA, USA), and King’s B medium (to test whether a strain produces green fluorescence visible upon UV exposure of 360–380 nm), respectively. Oxygen requirements were determined using thioglycollate medium (Hardy Diagnostics, CA. USA) [33]. A single, well-isolated colony of each strain was stab-inoculated to the bottom of the tube using a sterile inoculating needle, and tubes were incubated at 30 °C for 24–48 h. Motility was assessed by culturing the bacterium in a semi solid growth motility test medium containing tryptone, sodium chloride, and agar. The tobacco hypersensitive test of the isolated bacterial strains was conducted using three tobacco plants together with positive and negative controls. To check for the bacterial strains’ ability to produce pectolytic enzymes that cause potato soft rot, a pectolytic assay was done on flame-sterilized, peeled potato slices (3 replicates). Additionally, levan production was assessed in *Pseudomonas* strain 21TX0197^T^, using nutrient agar supplemented with 5% sucrose. Imaging was performed using cultures that were grown for 24 h at 30 °C in nutrient broth (per l, beef extract 3 g, peptone 5 g, sodium chloride 5 g) and visualized using calibrated magnification with a 15 C CCD camera on a JEOL 1200Ex transmission electron microscope (TEM) operated at 100 kV at the Texas A&M Microscopy and Imaging Centre following previously described protocols on negative staining [34].

For all three novel strains, physiological profiling was done using Biolog GEN III microplates. The Biolog GEN III assay relies on tetrazolium dye reduction to assess metabolic activity rather than direct growth. Therefore, stressed or dying cells may still generate positive reactions, potentially leading to false-positive interpretations of substrate utilization or growth capability. Hence, optimum growth conditions for the bacterial strains were ascertained via NaCl tolerance, pH tolerance, and optimum temperature tests. All three tolerance tests were conducted in triplicates using nutrient agar media with variable values for salt (0–10%), pH (4–10), and temperature (4–41 °C) [15]. Also, various API strips (bioMérieux) were utilized for selected strains and tested using manufacturer’s guidelines. API 20NE was used to characterize both the novel *Pseudomonas* and *Curtobacterium* strains. API ZYM was used to characterize both the novel *Curtobacterium* and *Falsirhodobacter* strains. While API 20E, API Coryne, and API 50CH were exclusively used for the novel *Curtobacterium* strain. For Biolog analysis, bacterial strains were cultured for 24-h-old in NA medium, suspended into inoculating fluid, adjusted for optical density (OD600) of 0.022, dispensed onto microplates according to the manufacturer’s instructions, and incubated at 30 °C to determine substrate utilization by bacteria [8]. The microplates were observed, and data were recorded after 24, 48, and 72 h. While some closely related strains were tested side by side in the lab, some data were used from previous studies, considering the similarity of the tested conditions.

## Results and Discussion

### Pathogenicity

Strain 21TX0197^T^, isolated from a rotting bulb, produced necrosis on scale, bulb, and foliage tissues (Fig. S1), whereas the other two strains—20TX0008^T^ and 20TX0035^T^—isolated from necrotic foliage were asymptomatic. On onion scales, after incubation for 4 days, necrotic lesions (~ 5 to 10 mm in width) with a pale-yellow appearance developed at the inoculation sites. While on onion bulbs, dark brown colored necrosis developed around the inoculated sites affecting over 30% of the bulb area at the dissected surface. Similarly, leaf showed dieback (~ 5–10 cm long) from the point of inoculation towards the bottom. Three strains with similar morphology across the tested pathogenicity assays were re-isolated for the symptomatic strain (GenBank accession of 16 S rRNA sequence: PV800730–PV800732). These daughter strains produced identical lesions during assays, thereby satisfying Koch’s postulates.

### 16 S rRNA Gene and Whole Genome Phylogeny

The strains 20TX0008^T^, 20TX0035^T^, and 21TX0197^T^ showed the highest 16S rRNA sequence similarities with *Curtobacterium caesinilyticum* GDMCC 1.2667^T^ (99.7%), *Falsirhodobacter deserti* W402^T^ (99.1%), and *Pseudomonas uvaldensis* 20TX0172^T^ (99.4%), respectively (Table 1). Although 16S rRNA gene similarity is often used for bacterial identification, even sequences with > 99.9% identity may belong to different species, and 16S-based species assignment should therefore be interpreted cautiously and be verified with the genome level analysis [32, 33]. In the phylogenetic trees based on 16S rRNA sequences, all three strains formed a distinct, well-supported branch that separated them from their closely related species within the same genus (Figures S2-S4).

**Fig. 1:**
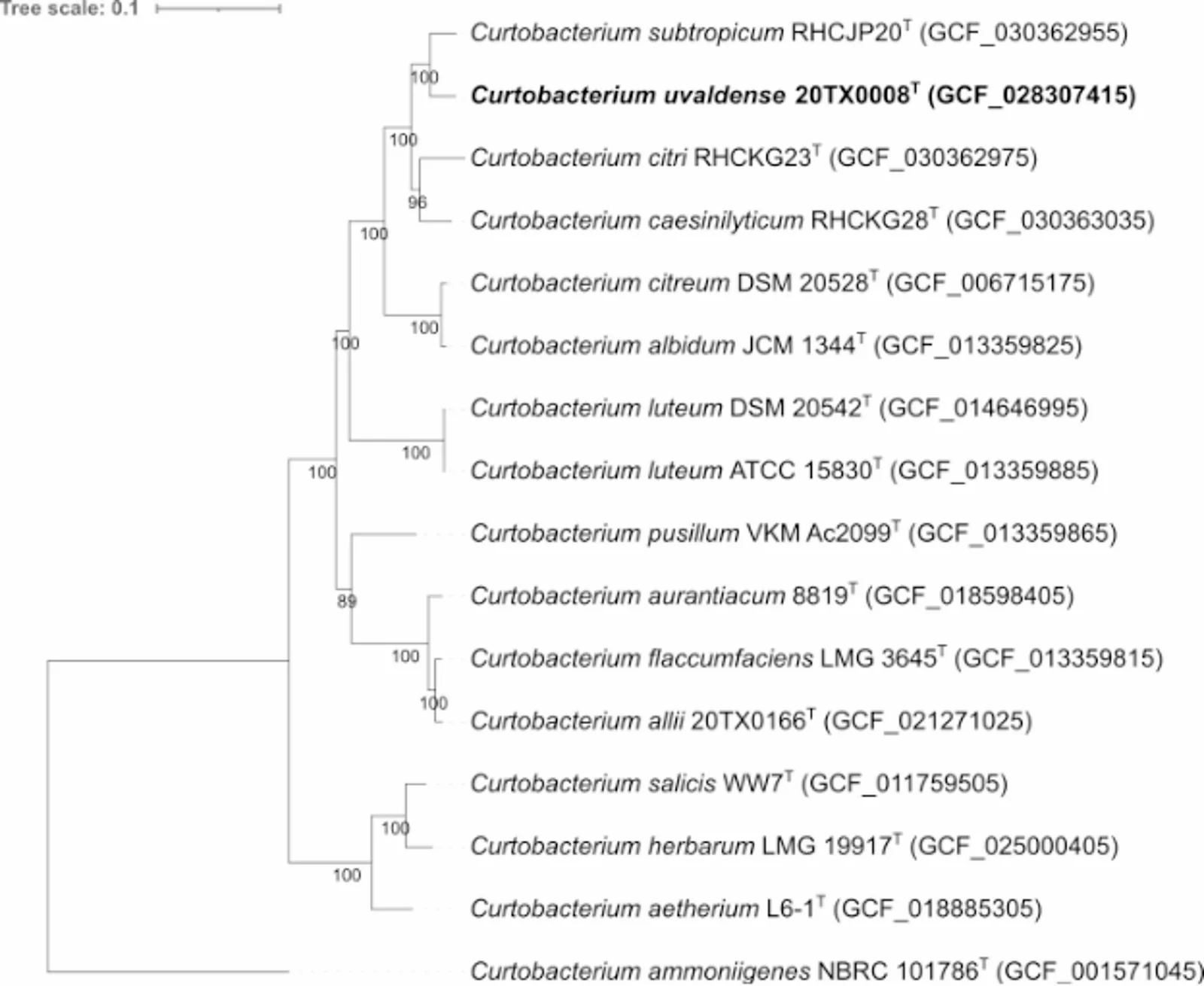
Phylogenetic tree inferred from 442 concatenated single‑copy core genes identified across the whole genome sequences of *Curtobacterium uvaldense* 20TX0008^T^ and closely related *Curtobacterium* species

**Fig. 2:**
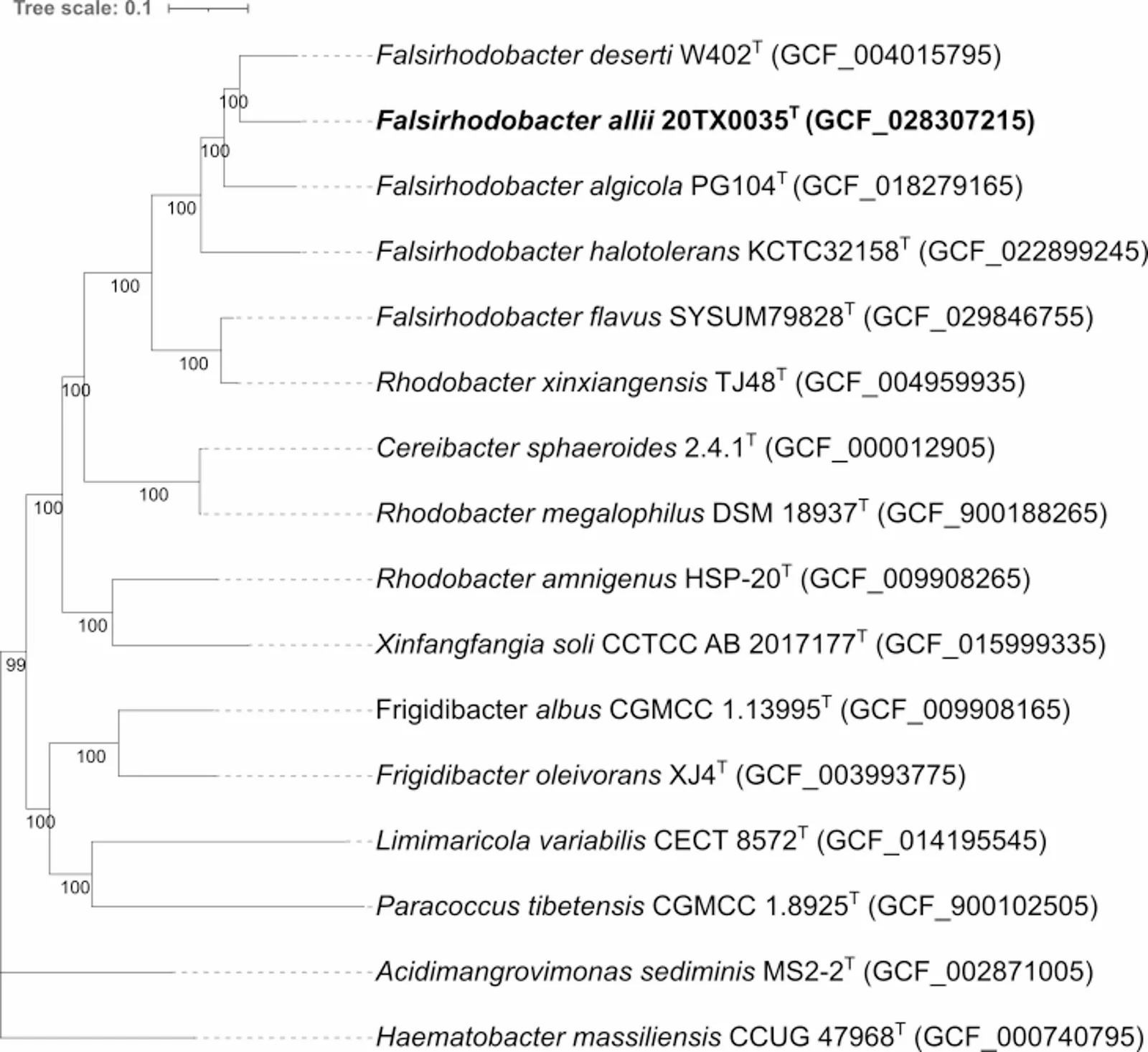
Phylogenetic tree inferred from 207 concatenated single‑copy core genes identified across the whole genome sequences of *Falsirhodobacter allii* 20TX0035^T^ and closely related *Falsirhodobacter* species

**Fig. 3:**
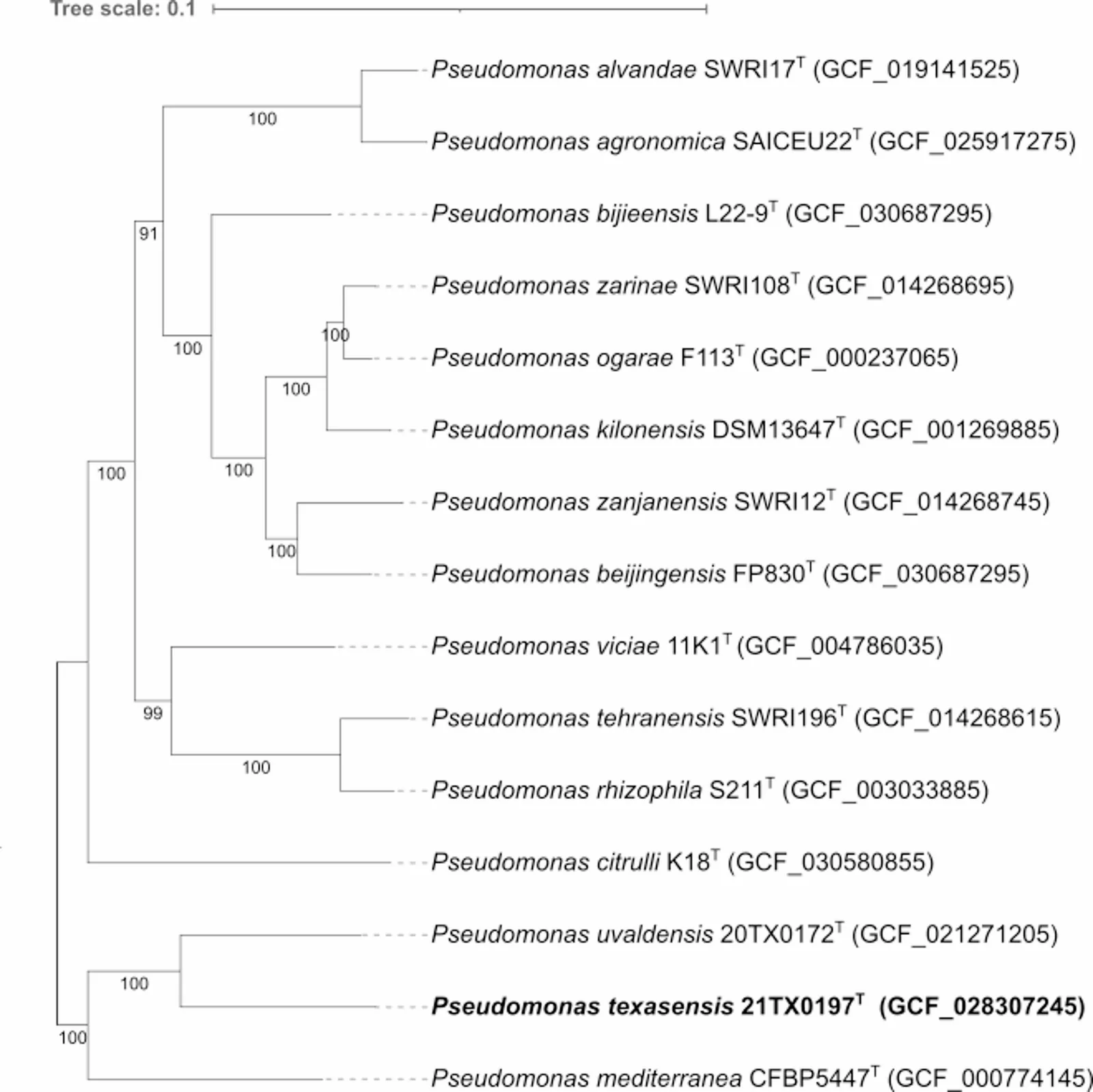
Phylogenetic tree inferred from 741 concatenated single‑copy core genes identified across the whole genome sequences of *Pseudomonas texasensis* 21TX0197^T^ and closely related *Pseudomonas* species

The whole genome analysis revealed that all isolated strains shared ANI values below 95% and dDDH values below 70% with their closest related species (Table 1), confirming their status as members of a distinct species. The in-silico dDDH values of strains 20TX0008^T^, 20TX0035^T^, and 21TX0197^T^ with their phylogenetically closest type strains — *Curtobacterium citri* GDMCC 1.2668^T^, *Falsirhodobacter deserti* W402^T^, and *Pseudomonas uvaldensis* 20TX0172^T^ — were estimated to be 40.0%, 23.3%, and 35.7%, respectively. The estimated ANIb values between strains 20TX0008ᵀ, 20TX0035ᵀ, and 21TX0197ᵀ and their respective closest type strains mentioned above were 86.8%, 79.8%, and 87.9%, respectively. The phylogenetic trees generated from concatenated single‑copy core gene markers (Figs. 1, 2 and 3) and from whole‑genome analyses using TYGS (Figures S5–S7) showed highly congruent topologies. In both approaches, the three strains consistently formed a distinct, well‑supported clade clearly separated from their closest described species within the genus, supporting their recognition as novel species.

### Genome Features and Functional Annotations

Among the three novel species, 21TX0197^T^ has the largest genome size (6.3 Mb, 100% completeness and 0.2% contamination), followed by 20TX0008^T^ (3.6 Mb, 100% completeness and 0.1% contamination) and 20TX0035^T^ (3.2 Mb, 98.8% completeness and 0.3% contamination). The estimated contig number in 20TX0008^T^ was 81 (N50 = 110.1 kb, L50 = 11 and G + C = 71.8%), in 20TX0035^T^ was 54 (N50 = 195 kb, L50 = 5 and G + C = 66.5%), and in 21TX0197^T^ was 130 (N50 = 198.5 kb, L50 = 8 and G + C = 61.5%). A total of 3,388 genes (3,314 protein-coding, 48 tRNA, and 3 rRNA) were found to be present in 20TX0008^T^, 3,276 genes (3,199 protein-coding, 45 tRNA, and 3 rRNA) in 20TX0035^T^, and 5,610 genes (5,475 protein-coding, 54 tRNA, and 4 rRNA) in 21TX0197^T^.

A comprehensive analysis of functional genomic annotations between the novel strains and the type strains of closely related species is provided in the Online Resource ESM_2 (Tables S1-S3). Also, a summary of the total, unique and shared gene roles across the strains is provided (Tables S4-S5). It is important to note that genome annotations reflect predicted gene content, and the presence of a gene does not guarantee its expression or functional activity. Likewise, genes not detected in the annotation may still be present but unrecognized due to limitations in current databases and prediction algorithms. The unique functional genomic potential of each novel strain is summarized below.

### *Curtobacterium* Strain 20TX0008^T^

Compared to nine closely related species, a total of four genes were uniquely identified in the novel strain 20TX0008^T^ (Table S1). These unique genes are each related to proline utilization, Type III restriction modification system, phase DNA synthesis, and beta-lactamase. Notably, the gene associated with class C beta-lactamase and penicillin-binding proteins (PBPs) was identified exclusively in this novel strain, with no homologous genes detected in any other strains analyzed. PBPs, essential for bacterial cell wall synthesis, also play significant roles in beta-lactam resistance mechanisms [35, 36]. Beta-lactamases, including Class C (AmpC) enzymes, are critical mediators of β-lactam antibiotic resistance [37, 38] and play a significant role in resistance to novel cephalosporins, with some species capable of inducible expression [39]. AmpC β-lactamases are typically chromosomally encoded in Gram-negative bacteria of the *Enterobacteriaceae* group, where their expression is often inducible or hyper-expressed due to promoter mutations. The “SPACE” organisms (*Serratia*, *Pseudomonas*, *Acinetobacter*, *Citrobacter*, *Enterobacter*) are clinically significant due to their chromosomal AmpC production [40]. While class C beta-lactamases are traditionally associated with Gram-negative bacteria, efficient class D beta-lactamases have been discovered in Gram-positive organisms (*Bacillaceae*, *Clostridiaceae*, and *Eubacteriaceae*), revealing a new reservoir of resistance enzymes [41]. The presence of this gene in the novel strain—a Gram-positive bacterium belonging to *Microbacteriaceae*—is particularly notable and unusual.

### *Falsirhodobacter* Strain 20TX0035^T^

Compared to six closely related species, a total of 18 genes were uniquely identified in the novel strain 20TX0035^T^, including seven associated with fructose utilization (Table S2). Notably, genes related to fructose utilization were absent in all other studied strains. Given that onion is a fructose-rich substrate [42, 43], the presence of multiple fructose utilization genes in the novel strain highlights a key adaptive trait for efficient nutrient acquisition and survival in its native environment. Although, the exact role of this gene is not identified in this strain, but, the ability to utilize D-fructose in Biolog assay could be linked to the presence of this gene. Similarly, only the novel strain possessed two genes, each involved in lignin degradation and orphan regulatory proteins—capabilities not detected in the related strains. A total of five genes related to niacin-choline transport and metabolism were identified. Of these, two were exclusively present in the novel strain, while the remaining three were shared only between *F. deserti* and the novel strain and were absent in all other studied strains.

### *Pseudomonas* Strain 21TX0197^T^

Compared to nine closely related species, a total of eight genes were uniquely identified in the novel strain 21TX0197^T^ (Table S3). Two genes involved in teichuronic acid biosynthesis—a component typically associated with Gram-positive cell walls—were uniquely identified in the novel strain, while such genes were absent in all other strains analyzed, making this an intriguing and unexpected finding. Teichuronic acids are important components of cell walls in some Gram-positive bacteria [44, 45]. While teichuronic acid biosynthesis genes are well-characterized in *Bacillus subtilis* [46, 47], their presence in the novel *Pseudomonas* strain, a Gram-negative species, highlights their exclusivity. The presence of these genes may endow the novel strain with the ability to synthesize teichuronic acid, potentially impacting its cell wall composition, environmental adaptability, or interactions with phosphate availability [45, 48].

Under subsystem “Toxin-antitoxin replicon stabilization systems”, two genes (YefM protein- antitoxin to YoeB, YoeB toxin protein) were found only in the novel strain 21TX0197^T^. The yefM-yoeB toxin-antitoxin (TA) system is often associated with plasmid and replicon stabilization, and plays important roles in cellular stress responses and biofilm formation [49]. In *Streptococcus*, deletion of yefM-yoeB reduces oxidative stress resistance and biofilm formation ability [50]. Two genes, Chromosome initiation inhibitor under subsystem LysR-family proteins *in Escherichia coli* and Universal stress protein family 4, under the stress response category, were found only in the novel strain. LysR-family proteins are among the most abundant transcriptional regulators in bacteria and play roles in metabolism, stress responses, and regulation of various cellular processes [51, 52]. The universal stress protein (USP) family is a superfamily of conserved proteins that are upregulated in response to diverse environmental stresses, including nutrient starvation, oxidative stress, and exposure to antibiotics or metals [53]. Under the Virulence, Disease, and Defense category, the cobalt-zinc-cadmium resistance pathway comprised a total of eight genes. Of these, seven were common to all strains, while the remaining one gene was uniquely present in the novel strain. Similarly, in the same category, a total of 15 genes were associated with copper homeostasis. Fourteen of these genes were shared across all strains, whereas one gene, linked to copper tolerance, was found exclusively in the novel strain. The presence of several unique stress-response genes underscores the novelty of the strain and suggests enhanced adaptability for survival.

An interesting pattern was observed in sulfur metabolism across nine *Pseudomonas* strains, comprising a total of 29 genes—17 associated with inorganic sulfur assimilation, eight with organic sulfur assimilation, and four with thioredoxin–disulfide reductase activity. All four thioredoxin–disulfide reductase genes were conserved across all strains. The novel strain, along with six others, contained only inorganic sulfur assimilation genes, lacking those for organic sulfur metabolism. In contrast, *P. mediterranea* exhibited the opposite trend—retaining genes solely for organic sulfur assimilation and lacking those for inorganic pathways. Notably, *P. uvaldensis*, the closest relative to the novel strain, was the only strain lacking both organic and inorganic sulfur assimilation genes. *P. bijieensis* was the only strain harboring genes for both pathways. Onions are known to accumulate inorganic sulfur compounds (e.g., sulfate, thiosulfate) as part of their defense and metabolic processes [54, 55]. The ability to efficiently assimilate inorganic sulfur could provide the novel strain with a competitive advantage in colonizing and infecting onion tissues.

### Physiological Features

The novel strain 20TX0008^T^ was Gram-stain positive, while the other two strains, 20TX0035^T^ and 21TX0197^T^, were Gram-stain negative. All the novel strains produced catalase and were non-endospore-forming, obligately aerobic, and non-pectolytic. The strain 20TX0008^T^ was non-motile, while the other two strains, 20TX0035^T^ and 21TX0197^T^, were motile with the presence of one or more flagella. For oxidase (production of cytochrome c oxidase), KB fluorescence, and tobacco hypersensitivity tests, all the strains responded negatively except 21TX0197^T^. The strain 21TX0197^T^ produced mucoid, dome-shaped colonies after 24 h at 25 °C on nutrient agar medium supplemented with 5% sucrose, which confirmed the production of levan, a fructan polysaccharide. TEM images showed that bacterial cells of all three novel strains were rod-shaped (Fig. S8). Cells of 20TX0008^T^ measured 1.15–2.10 μm long × 0.59–0.91 μm wide (mean ± SD, 1.55 ± 0.29 × 0.71 ± 0.09 μm; *n* = 10), cells of 20TX0035^T^ measured 1.47–2.39 μm long × 0.91–1.34 μm wide (mean ± SD, 1.93 ± 0.31 × 1.10 ± 0.14 μm; *n* = 10), and cells of 21TX0197^T^ measured 1.97–2.70 μm long × 0.78–0.99 μm wide (mean ± SD, 2.34 ± 0.25 × 0.90 ± 0.06 μm; *n* = 10). The growth conditions findings for all strains are presented in Tables 2, 3 and 4. Among *Curtobacterium* species, strain 20TX0008^T^ growth showed the highest tolerance to NaCl (up to 6%), alkaline conditions (up to pH 9), and the highest minimum temperature (15 °C) (Table 2). In contrast, among *Falsirhodobacter* species, strain 20TX0035^T^ exhibited the lowest NaCl tolerance (up to 3%) (Table 3). Similarly, within *Pseudomonas* species, strain 21TX0197^T^ displayed lower NaCl tolerance (up to 3%), compared to *P*. *uvaldensis*, which tolerated up to 4% NaCl (Table 4). An overview of the Biolog and API assays for each novel strain, highlighting the key reactions that distinguished the novel strains from the type strains of closely related species, is provided in Tables 2, 3 and 4. The comprehensive results of the Biolog and various API assays for the three novel strains are provided in Supplementary 2 (Tables S6-S9).

**Table 2:** Differential phenotypic characteristics of *Curtobacterium uvaldense* 20TX0008^T^ and closely related type strains of genus *Curtobacterium*. Strains: 1, 20TX0008^T^; 2, *C*. *subtropicum* RHCJP20^T^; 3, *C. citri* RHCKG23^T^; 4, *C. caesinilyticum* RHCKG28^T^. Data are from: λ, this study; ϕ, strains 2–4 from previous study [56]. −, negative reaction; +, positive reaction; w, weakly positive reaction

Characteristics	1 λ	2 ϕ	3 ϕ	4 ϕ
**Growth characteristics**				
NaCl tolerance (% w/v)	0–6	0–1	0–1	0–2
pH range	5–9 (optimum 6)	5–7 (optimum 6)	4–8 (optimum 6)	4–7 (optimum 6)
Temperature (°C) range	15–37 (optimum 30)	10–37 (optimum 28–30)	10–37 (optimum 28–30)	10–37 (optimum 28–30)
**Physiological characteristics**				
Motility	‒	+	+	+
Sources utilization (Biolog Gen III)*				
**Carbon sources**				
3-Methyl glucose	‒	+	‒	+
D-Arabitol	+	+	‒	+
D-Fucose	‒	‒	‒	+
D-Glucuronate	+	+	‒	+
D-Raffinose	+	+	‒	‒
D-Sorbitol	+	+	‒	+
Glucuronamide	w	+	‒	+
L-Fucose	‒	‒	‒	+
L-Galactonate lactone	‒	+	‒	+
L-Malate	w	+	‒	‒
L-Rhamnose	‒	‒	+	‒
**Enzyme activities (API ZYM)**				
*Amino peptidase*				
Cystine arylamidase	+	‒	+	w
Leucine arylamidase	+	‒	+	+
Valine arylamidase	+	‒	+	w
*Glycosyl hydrolase*				
N-Acetyl-β-glucosaminidase	+	+	+	‒
α-Galactosidase	+	w	+	+

*Substrate utilization was determined using Biolog GEN III microplates and indicates the ability to utilize compounds as sole carbon sources

**Table 3:** Differential phenotypic characteristics of *Falsirhodobacter allii* 20TX0035^T^ and closely related type strains of genus *Falsirhodobacter*. Strains: 1, 20TX0035^T^; 2, *F. deserti* strain W402^T^; 3, *F*. *algicola* PG 104^T^; 4, *F. halotolerans* KCTC 32158^T^. Data are from: λ, this study; ϕ, strains 2–4 from previous study [14]. −, negative reaction; +, positive reaction; w, weakly positive reaction

Characteristics	1 λ	2 ϕ	3 ϕ	4 ϕ
**Growth characteristics**				
NaCl tolerance (% w/v)	0–3	0–10	0–8	0–7
pH range	6–9 (optimum 7)	5.5–10 (optimum 7)	6–10 (optimum 7.5)	6-8.5 (optimum 7)
Temperature (°C) range	15–35 (optimum 30)	10–40 (optimum 30)	15–35 (optimum 30)	10–30 (optimum 30)
**Physiological characteristics**				
Motility	+	‒	+	+
Sources utilization (Biolog Gen III)*				
**Carbon sources**				
Bromo-succinate	+	+	+	‒
Citrate	‒	+	‒	+
D-Aspartate	‒	‒	‒	+
D-Cellobiose	‒	‒	+	‒
D-Fucose	w	+	‒	‒
D-Galacturonate	‒	+	‒	‒
D-Glucose-6-Phosphate	‒	‒	+	‒
D-Glucuronate	‒	+	‒	+
D-Maltose	‒	‒	+	‒
D-Mannose	+	+	‒	‒
D-Salicin	‒	‒	+	‒
D-Sorbitol	+	‒	‒	+
D-Trehalose	w	‒	+	‒
D-Turanose	‒	‒	+	‒
Glucuronamide	‒	+	‒	‒
L-Aspartate	+	‒	+	+
L-Pyroglutamate	+	+	‒	+
L-Rhamnose	‒	+	‒	‒
L-Serine	w	‒	‒	+
Methyl Pyruvate	+	‒	+	+
Tween 40	w	‒	+	‒
*α*-D-Glucose	+	+	‒	‒
**Chemical sensitivity (Biolog Gen III)**				
Lincomycin	+	+	‒	+
Nalidixic acid	+	+	+	‒
Rifamycin SV	‒	+	‒	+
**Enzyme activities (API ZYM)**				
*Amino peptidase*				
Valine arylamidase	+	‒	w	‒
*Esterase*				
Esterase (C4)	+	+	‒	‒
*Glycosyl hydrolase*				
N-acetyl-β-glucosaminidase	‒	‒	‒	+
β-galactosidase	‒	‒	w	‒
β-glucosidase	‒	‒	+	+
β-glucuronidase	‒	‒	+	‒
*Phosphatase*				
Acid phosphatase	+	w	+	‒
Naphthol-AS-BI-phosphohydrolase	+	‒	w	‒

*Substrate utilization was determined using Biolog GEN III microplates and indicates the ability to utilize compounds as sole carbon sources

**Table 4:** Differential phenotypic characteristics of *Pseudomonas texasensis* 21TX0197^T^ and closely related type strains of genus *Pseudomonas*. Strains: 1, 21TX0197^T^; 2, *P. uvaldensis* 20TX0172^T^; 3, *P*. *mediterranea* DSM 16733^T^. Data are from: λ, this study; ϕ, strains 2–3 from previous study [15]. −, negative reaction; +, positive reaction; w, weakly positive reaction; ND, no data available

Characteristics	1 λ	2 ϕ	3 ϕ
**Growth characteristics**			
NaCl tolerance (% w/v)	0–3	0–4	ND
pH range	5–9 (optimum 7)	5–10 (optimum 6–7)	ND
Temperature (°C) range	4–41 (optimum 30)	4–41 (optimum 25–30)	ND
Sources utilization (Biolog Gen III)*			
**Carbon sources**			
D-Fructose-6-Phosphate	‒	w	‒
D-Fucose	w	+	+
D-Galacturonate	w	‒	+
D-Saccharate	‒	+	+
D-Trehalose	‒	+	+
Formate	w	‒	+
Glucuronamide	w	+	+
Inosine	w	+	+
L-Fucose	‒	w	w
L-Galactonate lactone	w	‒	+
L-Histidine	w	+	+
p-Hydroxy-phenyl-acetate	‒	‒	+
Sucrose	‒	+	+
Tween 40	w	‒	‒
*α*-Hydroxy-butyric acid	w	w	+
**Chemical sensitivity (Biolog Gen III)**			
Sodium bromate	+	+	‒
**Enzyme activity (API 20NE)**			
*Amino peptidase*			
Arginine dihydrolase	+	+	‒

*Substrate utilization was determined using Biolog GEN III microplates and indicates the ability to utilize compounds as sole carbon sources

## Conclusion

Taken together, the phylogenetic placement, genome‑wide relatedness indices (ANI < 91%, dDDH < 41%), and distinct physiological and functional characteristics clearly demonstrate that strains 20TX0008ᵀ, 20TX0035ᵀ, and 21TX0197ᵀ each represent well‑separated, previously uncharacterized species within their respective genera. Their unique genomic features, combined with diagnostic phenotypic traits that differentiate them from the closest type strains, provide strong and consistent evidence supporting the establishment of three novel species.

### Description of *Curtobacterium uvaldense* sp. nov

*Curtobacterium uvaldense* (u.val.denʹse. N.L. neut. adj. *uvaldense*, pertaining to Uvalde, Texas, USA, where the type strain was isolated).

Cells are Gram-stain-positive, obligate aerobe, rod-shaped, 1.1–2.1 μm long × 0.6–0.9 μm wide, non-spore-forming and non-motile. Produce catalase but not cytochrome c oxidase. Colonies on nutrient agar are pale yellow, circular, entire, smooth, raised and 0.5–1.0 mm in diameter after 24 h at 25 °C. Growth occurs at 15–37 °C, with an optimum at 30 °C, and at pH 5.0–9.0, with an optimum at pH 6.0. Growth occurs on nutrient agar medium in the presence of 0–6% (w/v) NaCl, with an optimum at 0% (w/v). Does not assimilate 3-methyl glucose, D-aspartate, D-fructose 6-phosphate, D-fucose, D-galacturonate, D-glucose 6-phosphate, D-lactate methyl ester, D-saccharate, D-serine, formate, γ-aminobutyrate, L-fucose, L-galactonate lactone, L-histidine, L-lactate, L-pyroglutamate, L-rhamnose, methyl pyruvate, mucate, N-acetyl-β-D-mannosamine, N-acetyl neuraminate, p-hydroxyphenylacetate, propionate, quinate, α-hydroxybutyrate, α-ketobutyrate, α-ketoglutarate or β-hydroxy-DL-butyrate as carbon sources. Weakly assimilates bromo‑succinate, citrate, D‑malate, glucuronamide or L‑malate as carbon sources.

The type strain 20TX0008^T^ (= LMG 33207^T^=NCIMB 15480^T^) was isolated from symptomatic onion foliage collected in Texas, USA. The genome size of the type strain is 3.6 Mbp and its DNA G + C content is 71.8%. The GenBank accession numbers for the genome sequence and 16 S rRNA sequence are JAQMHU000000000 and ON255719, respectively. The type strain does not grow in the presence of D‑serine, fusidic acid, lincomycin, minocycline, niaproof 4, rifamycin SV, tetrazolium blue, tetrazolium violet, troleandomycin, and vancomycin. And it exhibits weak sensitivity to guanidine HCl and sodium bromate. Under the conditions tested, the type strain does not cause necrosis in inoculated onion tissues, does not produce a hypersensitive response in tobacco leaves, and has no pectolytic activity on potato slices.

### Description of *Falsirhodobacter allii* sp. nov

*Falsirhodobacter allii* (alʹli.i. L. gen. n. *allii*, of onion, *Allium cepa*, referring to the source from which the type strain was isolated).

Cells are Gram-stain-negative, obligate aerobe, rod-shaped, 1.5–2.4 μm long × 0.9–1.3 μm wide, non-spore-forming and motile. Produce catalase but not cytochrome c oxidase. Colonies on nutrient agar are yellow, circular, entire, smooth, flat and 0.5–1.0 mm in diameter after 24 h at 25 °C. Growth occurs at 15–35 °C, with an optimum at 30 °C, and at pH 6.0 to 9.0, with an optimum at pH 7.0. Growth occurs on nutrient agar medium in the presence of 0–3% (w/v) NaCl, with an optimum at 0% (w/v). Assimilates acetate, acetoacetate, bromo-succinate, D-arabitol, D-fructose, D-galactose, D-malate, D-mannitol, D-mannose, D-sorbitol, glycerol, L-alanine, L-aspartate, L-glutamate, L-malate, L-pyroglutamate, methyl pyruvate, α-D-glucose, α-hydroxybutyrate and α-ketoglutarate as sole carbon sources. Weakly assimilates D-fucose, D-lactic acid methyl ester, D-saccharic acid, D-trehalose, glycyl-L-proline, L-serine or Tween 40 as carbon sources.

The type strain 20TX0035^T^ (= LMG 33208^T^=NCIMB 15477^T^) was isolated from symptomatic onion foliage collected in Texas, USA. The genome size of the type strain is 3.2 Mbp and its DNA G + C content is 66.5%. The GenBank accession numbers for the genome sequence and 16 S rRNA gene sequence are JAQMHV000000000 and ON255745, respectively. The type strain grows in the presence of lincomycin, lithium chloride, nalidixic acid, and 1% sodium lactate. Under the conditions tested, the type strain does not cause necrosis in inoculated onion tissues, does not produce a hypersensitive response in tobacco leaves, and has no pectolytic activity on potato slices.

### Description of *Pseudomonas texasensis* sp. nov

*Pseudomonas texasensis* (tex.as.enʹsis. N.L. fem. adj. *texasensis*, pertaining to Texas, USA, where the type strain was isolated).

Cells are Gram-stain-negative, obligate aerobe, rod-shaped, 2.0–2.7 μm long × 0.8–1.0 μm wide, non-spore-forming and motile. Produce catalase and cytochrome c oxidase. Colonies on nutrient agar are cream-coloured, circular, entire, smooth, raised, glossy and 0.5–1.0 mm in diameter after 24 h at 25 °C. Produce a light green diffusible pigment on King’s B agar and fluoresce green under near-ultraviolet illumination at 360–380 nm. Produce levan on nutrient agar supplemented with 5% sucrose. Growth occurs at 4–41 °C, with an optimum at 30 °C, and at pH 5.0 to 9.0, with an optimum at pH 7.0. Growth occurs on nutrient agar medium in the presence of 0–3% (w/v) NaCl, with an optimum at 0% (w/v). Does not assimilate 3-methyl glucose, acetoacetate, bromo-succinate, D-aspartate, D-cellobiose, D-fructose 6-phosphate, D-glucose 6-phosphate, D-glucuronate, D-lactate methyl ester, D-malate, D-maltose, D-melibiose, D-raffinose, D-saccharate, D-salicin, D-serine, D-sorbitol, D-trehalose, D-turanose, dextrin, gelatin, gentiobiose, glycyl-L-proline, L-fucose, L-rhamnose, N-acetyl-D-galactosamine, N-acetyl-β-D-mannosamine, N-acetylneuraminate, pectin, p-hydroxyphenylacetate, stachyose, sucrose, α-D-lactose or β-methyl-D-glucoside as carbon sources. Weakly assimilates D-fucose, D-galacturonate, formate, glucuronamide, inosine, L-galactonate lactone, L-histidine, tween 40, α-hydroxybutyrate or α-ketobutyrate as carbon sources.

The type strain 21TX0197^T^ (= LMG 33210^T^=NCIMB 15479^T^) was isolated from a symptomatic onion bulb collected in Texas, USA. The genome size of the type strain is 6.3 Mbp and its DNA G + C is 61.5%. The GenBank accession numbers for the genome sequence and 16 S rRNA gene sequence are JAQMHS000000000 and OP325568, respectively. The type strain does not grow in the presence of D‑serine, and sodium butyrate. Under the conditions tested, the type strain causes necrosis in inoculated onion tissues, produces a hypersensitive response in tobacco leaves, but has no pectolytic activity on potato slices.

## Supplementary Information

Below is the link to the electronic supplementary material.

Supplementary Material 1

Supplementary Material 2

## Data Availability

All data supporting the findings of this study are available within the paper and its Supplementary Information.Genome sequence data were deposited into the GenBank database. The GenBank accession numbers for the genomes of strains 20TX0008^T^, 20TX0035^T^, and 21TX0197^T^ are NZ_JAQMHU000000000 (https://www.ncbi.nlm.nih.gov/nuccore/NZ_JAQMHU000000000), NZ_JAQMHV000000000 (https://www.ncbi.nlm.nih.gov/nuccore/NZ_JAQMHV000000000), and NZ_JAQMHS000000000 (https://www.ncbi.nlm.nih.gov/nuccore/NZ_JAQMHS000000000), respectively.

## Abbreviations

ANI: Average nucleotide identity
dDDH: digital DNA-DNA hybridization
ICNP: International Code of Nomenclature of Prokaryotes
LPSN: List of Prokaryotic names with Standing in Nomenclature
NA: Nutrient agar
BLAST: basic local alignment search tool
NCBI: National Center for Biotechnology Information
orthoANIu: orthologous ANI
ANIb: ANI based on BLAST
TYGS: Type Strain Genome Server
OD: optical density
RAST: Rapid Annotation using Subsystem Technology

## Declarations

### Authors’ statements

The authors used AI tools, including ChatGPT (OpenAI, GPT-5), Microsoft Copilot, Elicit, and Perplexity, to support manuscript preparation and literature review. These tools were employed for writing refinement, figure formatting, and rapid summarization of relevant studies. All scientific claims and interpretations were independently verified against primary sources, and full responsibility for the content rests solely with the authors.

### Repositories

The GenBank accession numbers for the genomes of strains 20TX0008^T^, 20TX0035^T^, and 21TX0197^T^ are JAQMHU000000000, JAQMHV000000000, and JAQMHS000000000, respectively. The GenBank accession numbers for the 16S rRNA sequences of strains 20TX0008^T^, 20TX0035^T^, and 21TX0197^T^ are ON255719, ON255745, and OP325568, respectively.
