# Supplementary material for: Curtobacterium uvaldense sp. nov., Falsirhodobacter allii sp. nov., Pseudomonas texasensis sp. nov., Three Novel Species Isolated from Onions in Texas, USA
Source: Curr Microbiol. 2026 Sep 9;83(11):559. doi: 10.1007/s00284-026-05144-1 (PMC13558446; doi:10.1007/s00284-026-05144-1)

***Curtobacterium uvaldense* sp. nov., *Falsirhodobacter allii* sp. nov., *Pseudomonas texasensis* sp. nov., three novel species isolated from onions in Texas, USA**

Author names

Manzeal Khanal[1,2‡], Bed Prakash Bhatta[1,2†], Lakhvir Kaur[1,2] and Subas Malla[1,2,*]

Affiliation

[1]Department of Horticultural Sciences, Texas A&M University, College Station, TX 77843, USA; [2]Texas A&M AgriLife Research and Extension Center, Uvalde, TX 78801, USA;

[‡]Current address: Department of Environmental Science, University of Arizona, Tucson, AZ 85719, USA; [†]Current address: Department of Agriculture, Alcorn State University, MS 39096, USA.

[*] Corresponding author: Subas Malla, subas.malla@ag.tamu.edu

Supplementary Fig. S1. Necrosis observed on (a) red onion scales 4 days post-inoculation, (b) onion bulb 12 days post-inoculation, and (c) onion foliage 5 days post-inoculation, with strain 21TX0197^{T} of *Pseudomonas* isolated from a rotten onion bulb collected in Texas, USA

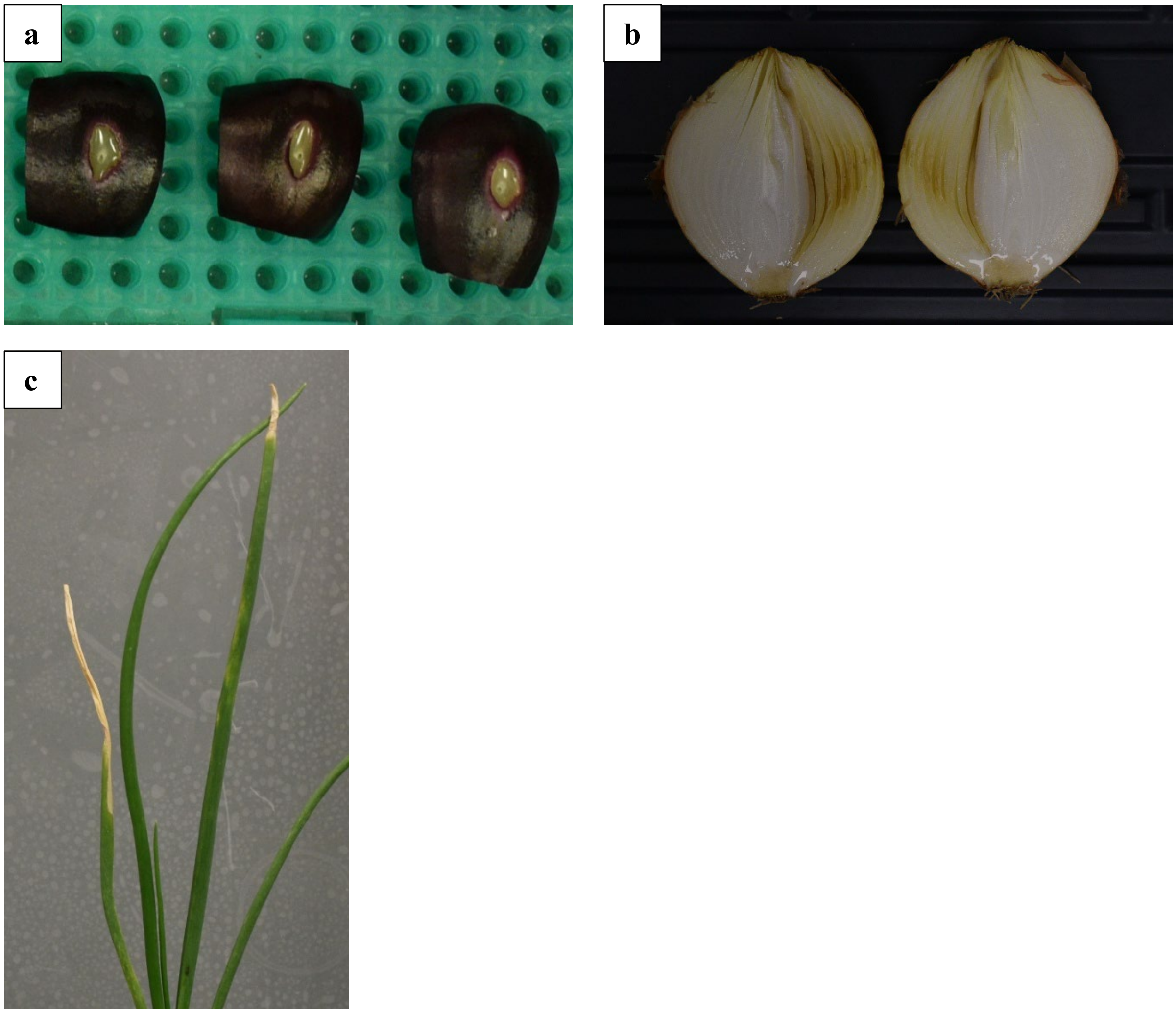

Supplementary Fig. S2. Phylogenetic tree based on 16S rRNA gene sequences showing the position of *Curtobacterium uvaldense* 20TX0008^{T} and closely related species of *Curtobacterium*

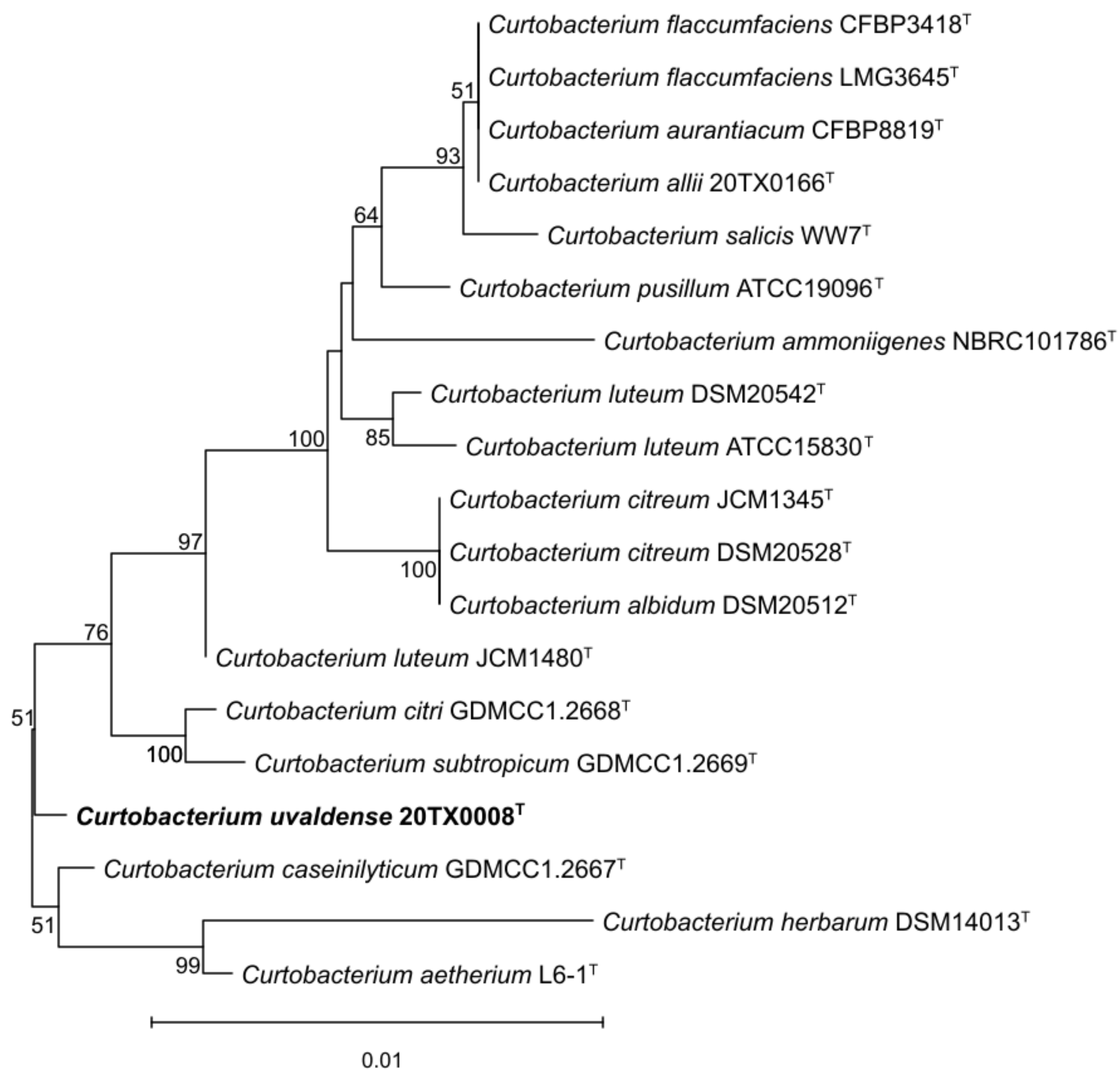

Supplementary Fig. S3. Phylogenetic tree based on 16S rRNA gene sequences showing the position of *Falsirhodobacter allii* 20TX0035^T and closely related species of *Falsirhodobacter*

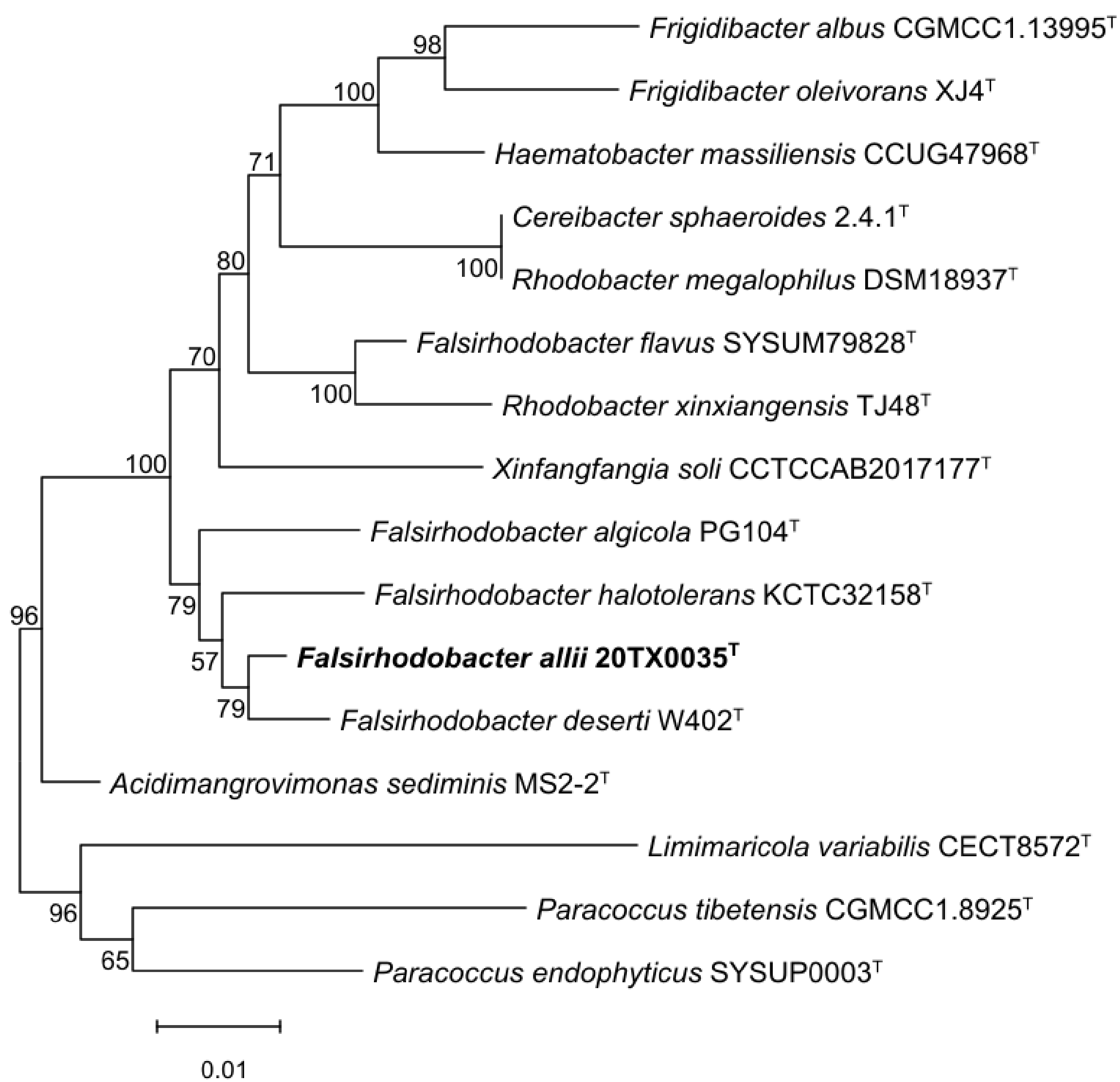

Supplementary Fig. S4. Phylogenetic tree based on 16S rRNA gene sequences showing the position of *Pseudomonas texasensis* 21TX0197^T and closely related species of *Pseudomonas*

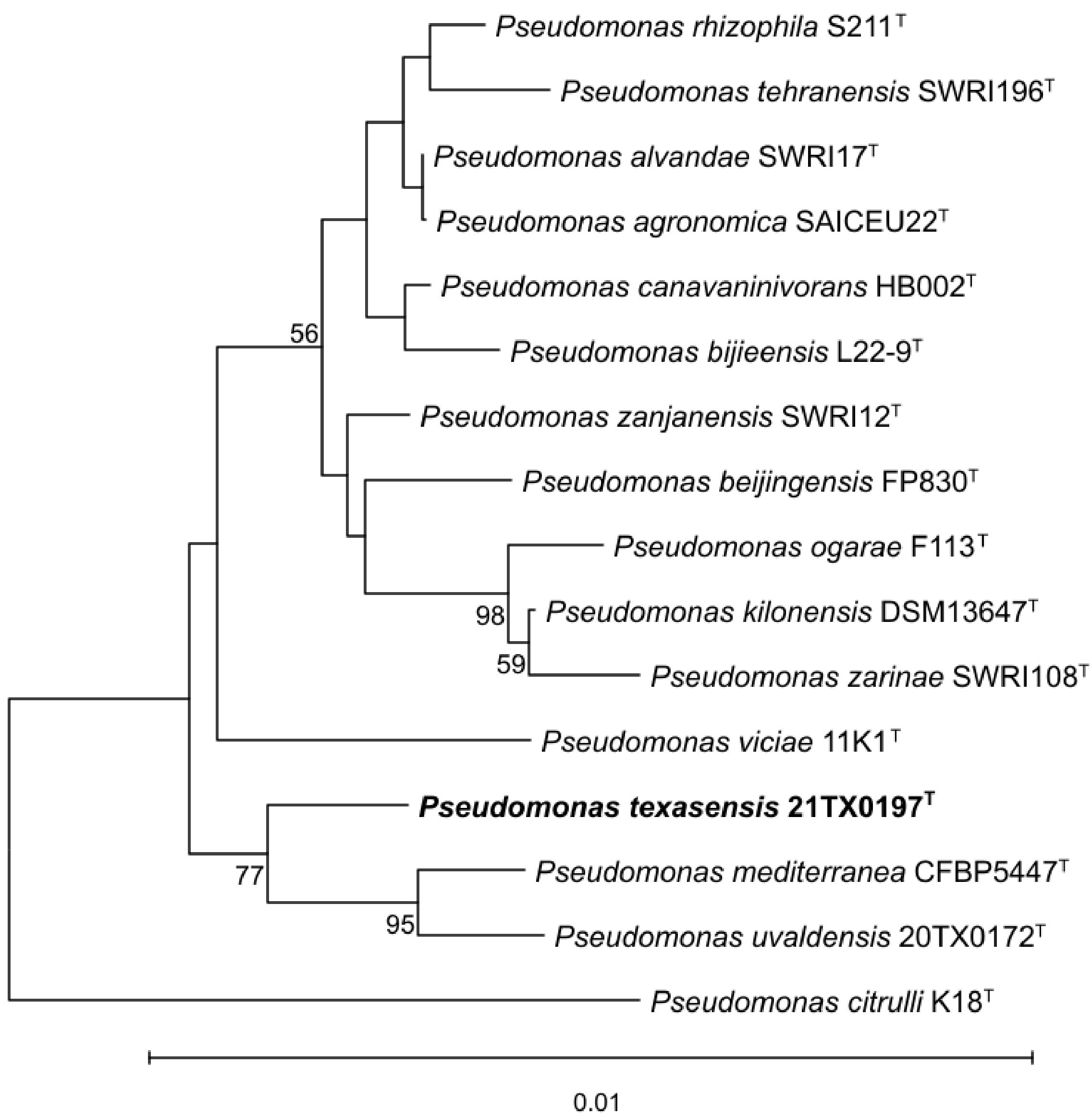

Supplementary Fig. S5. Phylogenetic tree constructed using TYGS, based on whole genome sequences showing the position of *Curtobacterium uvaldense* 20TX0008^{T} and closely related species of *Curtobacterium*

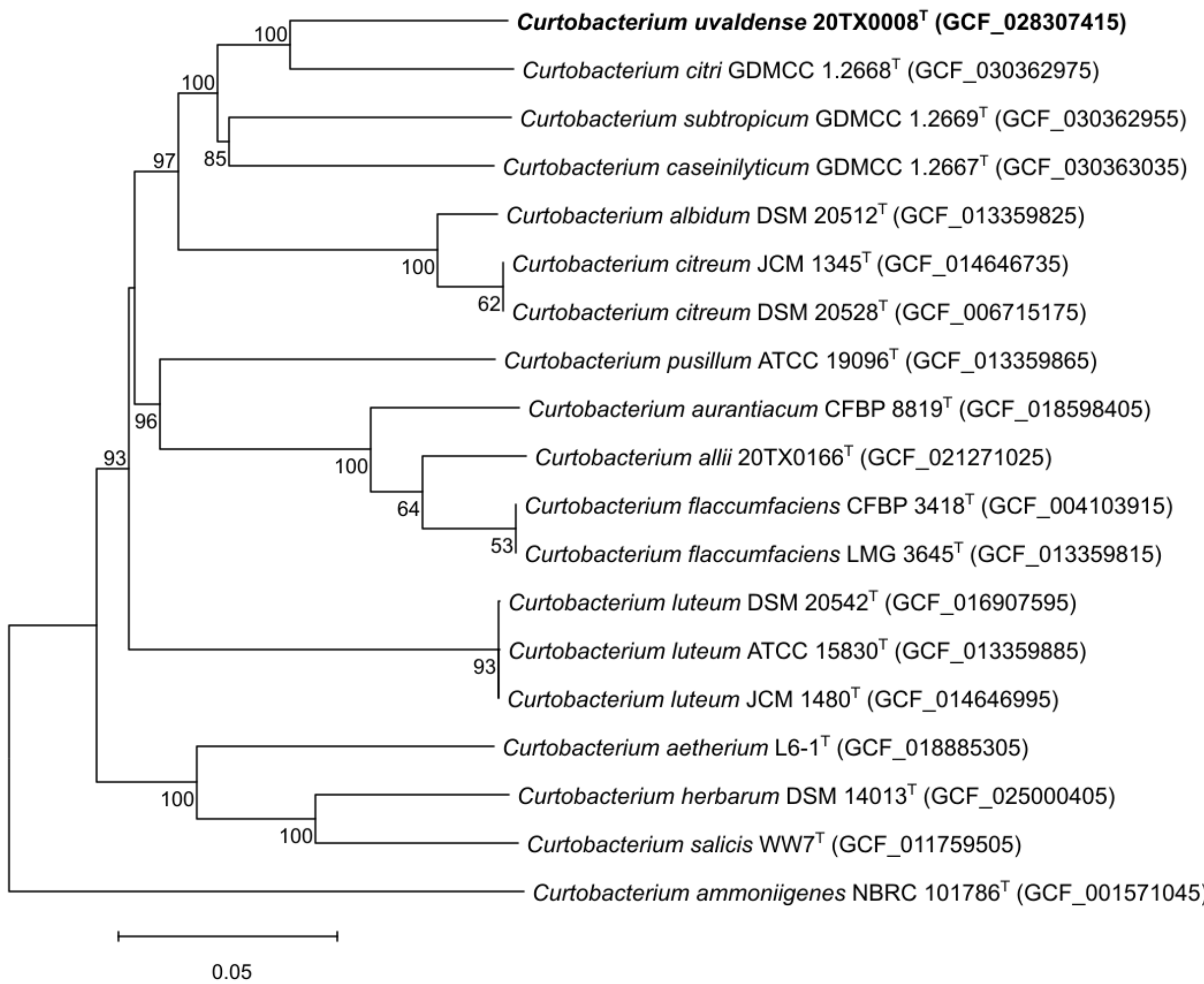

Supplementary Fig. S6. Phylogenetic tree constructed using TYGS, based on whole genome sequences showing the position of *Falsirhodobacter allii* 20TX0035^T and closely related species of *Falsirhodobacter*

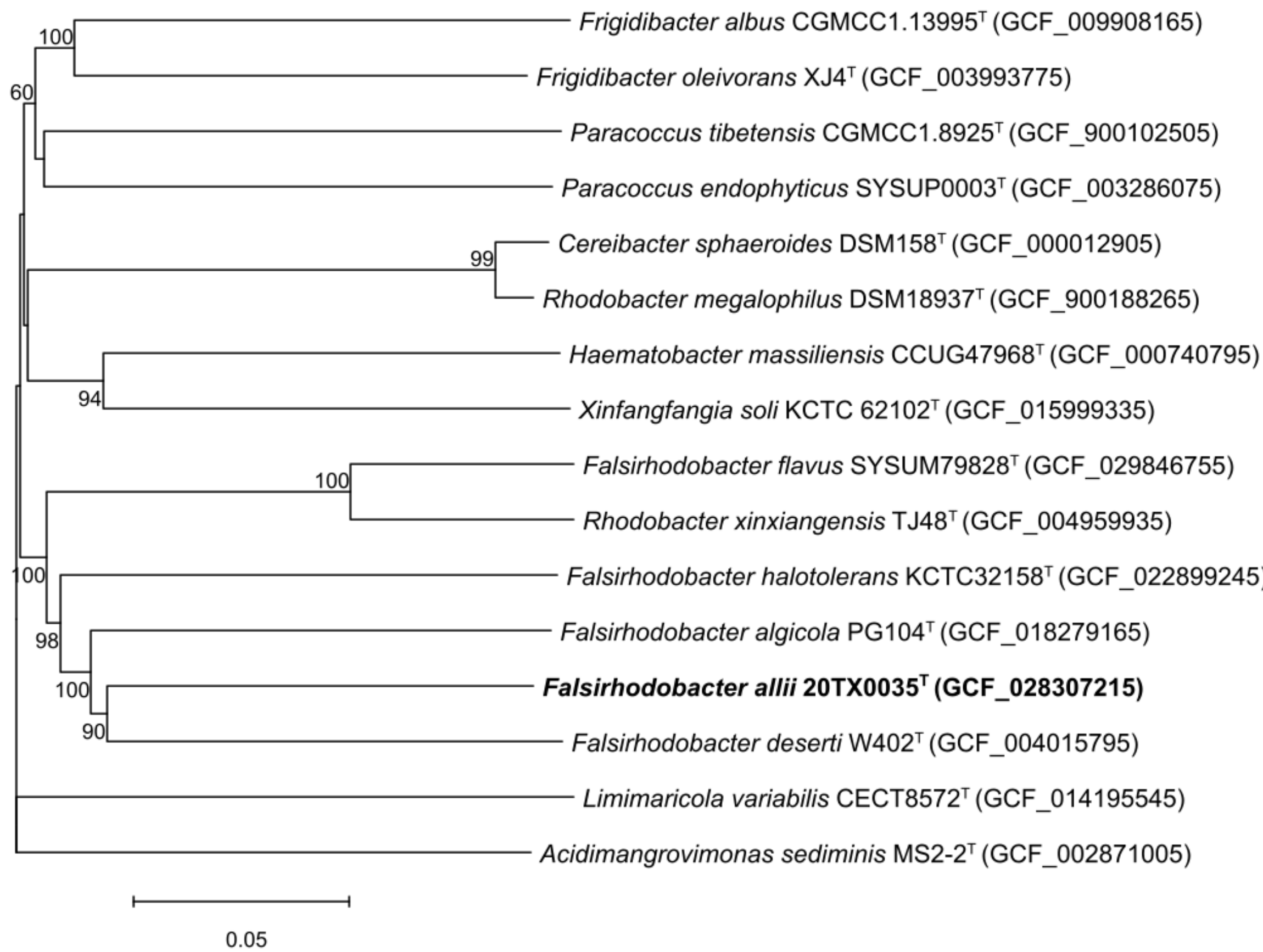

Supplementary Fig. S7. Phylogenetic tree constructed using TYGS, based on whole genome sequences showing the position of *Pseudomonas texasensis* 21TX0197^T and closely related species of *Pseudomonas*

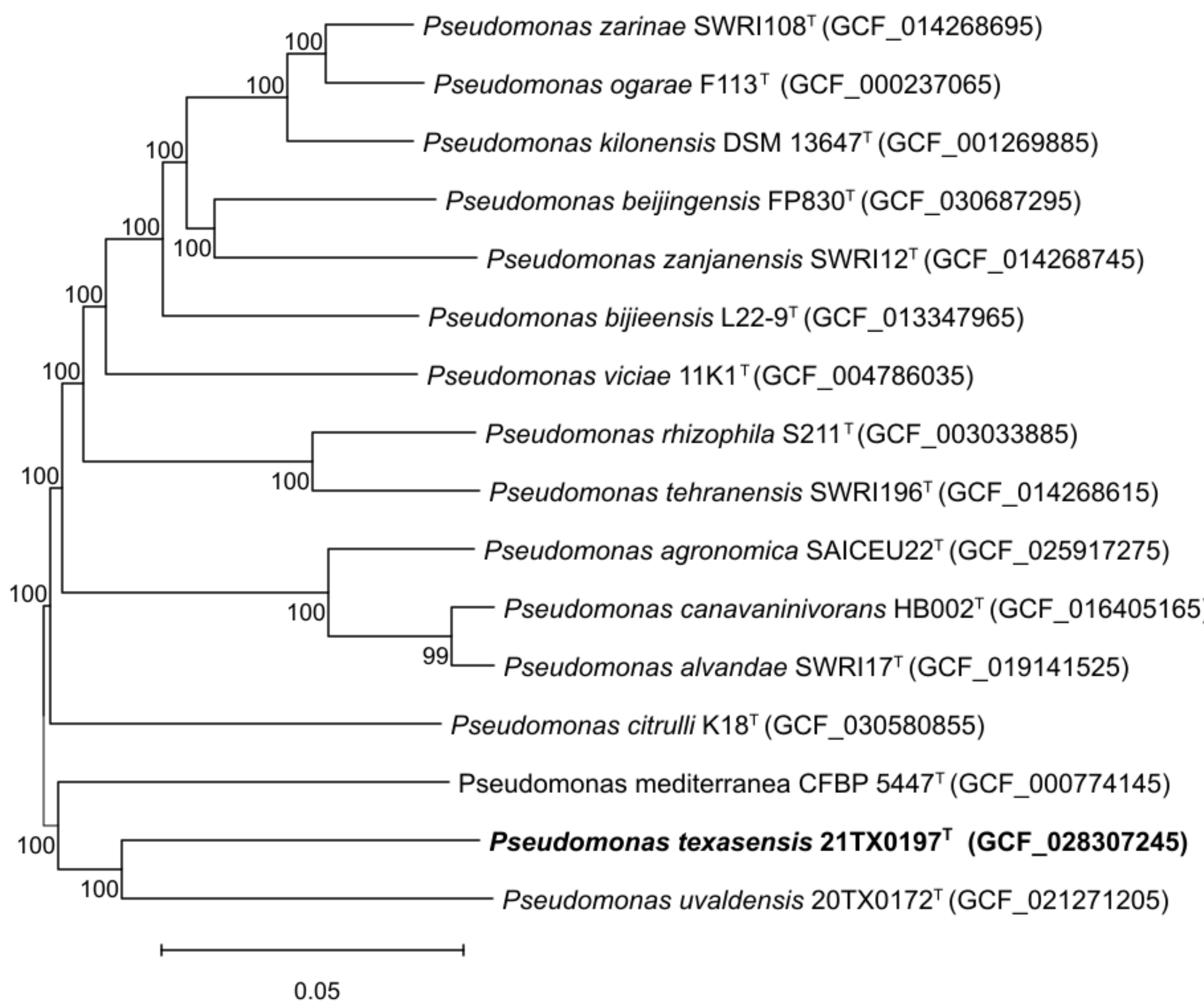

Supplementary Fig. S8. Transmission electron micrographs of cells from the novel strains: (a) *Curtobacterium uvaldense* 20TX0008^{T}, (b) *Falsirhodobacter allii* 20TX0035^{T}, and (c) *Pseudomonas texasensis* 21TX0197^{T}. Scale bars represent 1 μm.

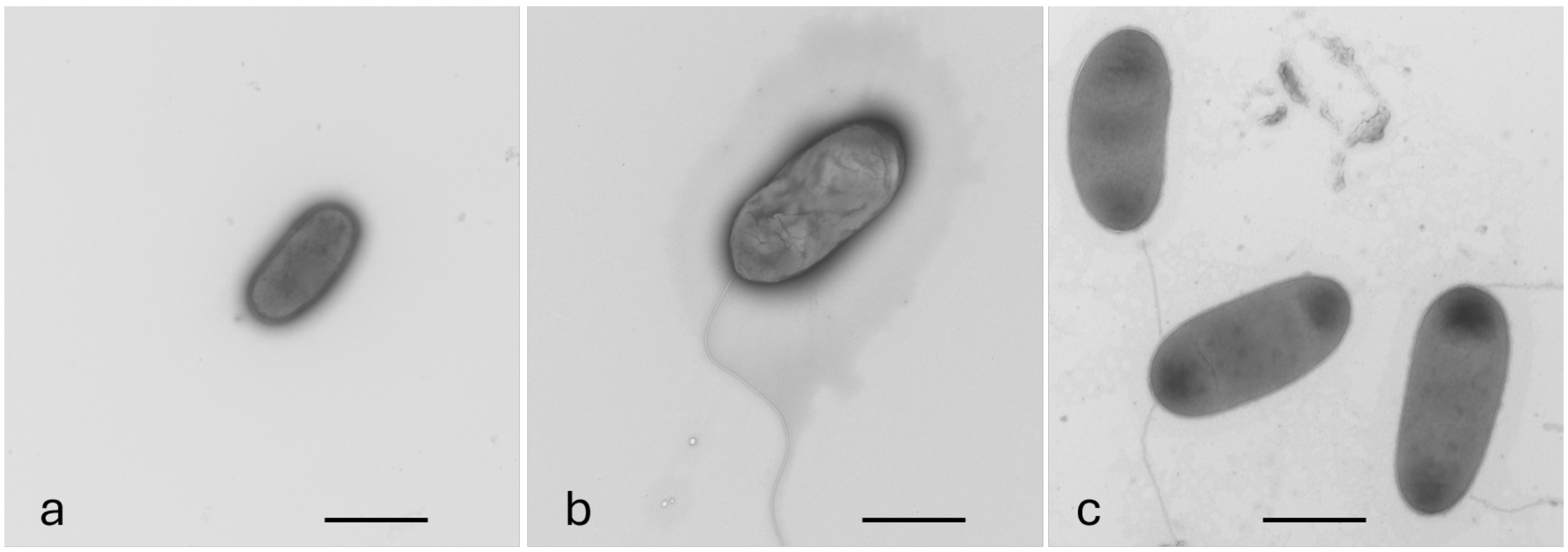

Supplement: Supplementary file 1 [file 284_2026_5144_MOESM1_ESM.pdf]
